# Chronological outcomes of renal function after adrenalectomy in patients with primary aldosteronism across age groups

**DOI:** 10.3389/fendo.2024.1467742

**Published:** 2024-11-07

**Authors:** Yu Ma, Xiaofeng Tang, Qian Ge, Jianzhong Xu, Pingjin Gao, Jiguang Wang, Limin Zhu

**Affiliations:** ^1^ Department of Cardiovascular Medicine, State Key Laboratory of Medical Genomics, Shanghai Key Laboratory of Hypertension, Shanghai Institute of Hypertension, Shanghai, China; ^2^ Department of Hypertension, Ruijin Hospital, Shanghai Jiao Tong University School of Medicine, Shanghai, China

**Keywords:** primary aldosteronism, age, adrenalectomy, estimated glomerular filtration rate, *KCNJ5* mutation

## Abstract

**Background:**

Patients with primary aldosteronism present with renal function decline after unilateral adrenalectomies. Our study aimed to assess the evolution of renal function after adrenalectomy in patients with primary aldosteronism across different age groups and to identify risk factors for postoperative renal function deterioration.

**Methods:**

We included 210 patients with primary aldosteronism categorized into three age groups: <40, 40–60, and ≥60 years old. We followed up the patients for 1 month, 1 year, and 5 years after adrenalectomy to assess outcomes. Multivariate analyses were performed to identify predictors of renal function deterioration, and a univariate logistic regression analysis was used to assess the relationship between *KCNJ5* mutation status and the decline in renal function.

**Results:**

Patients aged <40 years had a shorter duration of hypertension, higher preoperative diastolic blood pressure, and higher preoperative estimated glomerular filtration rate (eGFR) than did those in the other age groups. This group also exhibited the highest rate of complete clinical success, although there were no significant differences in complete biochemical success among age groups. Renal function declined in all three groups after adrenalectomy. However, changes in blood pressure and eGFR in the short- or long-term after adrenalectomy showed no significant differences among the three groups. Hypertension duration, preoperative systolic blood pressure (SBP), and plasma aldosterone concentration (PAC) were predictors of postoperative renal function deterioration. *KCNJ5* wild-type status was significantly correlated with the occurrence of chronic kidney disease after adrenalectomy.

**Conclusions:**

Unilateral adrenalectomy demonstrates favorable biochemical and clinical outcomes in patients with primary aldosteronism, irrespective of age. Long-term eGFR decline is similar among the different age groups. *KCNJ5* mutation exhibits a protective effect against the risk of chronic kidney disease after unilateral adrenalectomy.

## Introduction

Primary aldosteronism (PA), characterized by inappropriately elevated aldosterone secretion, represents the most prevalent endocrine cause of secondary hypertension, affecting approximately 5–10% of patients with hypertension (1, 2). Excess aldosterone causes hypertension and hypokalemia, increasing the risk of cardiovascular and kidney diseases in PA (3–5).

Surgical adrenalectomy or mineralocorticoid receptor antagonists (MRAs) are the two recommended therapies, and the treatment choice is based on PA subtype and patient’s preference (6, 7). Surgical treatment may resolve the excessive aldosterone production in patients with PA. A meta-analysis including 43 studies involving approximately 4000 patients with PA reported that the hypertension cure rate was approximately 50.6% for unilateral adrenalectomy (8).

Excess cardiovascular and cerebrovascular events and renal dysfunction in PA patients are ameliorated after adrenalectomy (4, 9–11). However, a significant proportion of patients develop renal dysfunction after adrenalectomy (12–15). For instance, Iwakura et al. (14) found that the prevalence of chronic kidney disease (CKD), defined as estimated glomerular filtration rate (eGFR) <60 mL/min/1.73 m^2^, increased from 15.7% before adrenalectomy to 37.1% at 12 months post-surgery in the aldosterone-producing adenoma (APA) group. Our clinical observations also noted that certain older patients with PA experienced declining eGFR or developed CKD after adrenalectomy. Consequently, preserving renal function has become a critical consideration, in addition to blood pressure (BP) management, in patients with PA undergoing adrenalectomy.

Whether older patients benefit from adrenalectomy in terms of renal function needs further studies.

Recent studies have emphasized the vital role of somatic mutations in the *KCNJ5* gene in the pathogenesis of APAs. The prevalence of *KCNJ5* somatic mutations in APAs is notably higher in Chinese patients (70.7%–80.7%) (16–18) than in patients from Western countries (34%) (19). Arnesen et al. (20) demonstrated that the presence of *KCNJ5* mutations was related to better surgical outcomes in patients with APAs, including lower postoperative BP and a higher prevalence of clinical cure. Zhang et al. (18) further reported that *KCNJ5* mutation was a protective factor for achieving complete clinical success. However, studies on the relationship between *KCNJ5* mutation status and postoperative renal function changes are limited.

In this study, we aimed to investigate the chronological changes in renal function in patients with PA of different ages and explore the predictive factors of postoperative renal deterioration after adrenalectomy in the Chinese population. Additionally, we aimed to evaluate the potential benefits of unilateral adrenalectomy especially in elderly patients.

## Methods

### Patients

We recruited 218 patients diagnosed with PA who consecutively underwent unilateral adrenalectomy at the Department of Hypertension of Ruijin Hospital between April 2010 and June 2019. Finally, we included 210 patients with *KCNJ5* mutations and wild-type (WT) variants at postoperative follow-up.

PA was diagnosed according to the 2016 Endocrine Society PA management guideline (6). Briefly, before the diagnostic workup, angiotensin-converting enzyme inhibitors (ACEis), angiotensin II type 1 receptor blockers (ARBs) and β-blockers were withdrawn for at least 2 weeks, non-potassium-sparing diuretics for 4 weeks, and MRAs for 6 weeks. Non-dihydropyridine calcium channel blockers and α_1_ blockers were prescribed for BP control, as necessary. Patients with a positive aldosterone-to-renin ratio (ARR) (ARR>24 [ng/dL]/[ng/mL/h]) were considered PA candidates. PA was confirmed using an intravenous saline loading infusion test (post-test aldosterone levels >100 pg/mL). The patients diagnosed PA underwent adrenal computed tomography scans. Adrenal venous sampling (AVS) was carried out in patients willing to undergo unilateral adrenalectomy. We conducted AVS by sequential procedure without ACTH stimulation and the selectivity and lateralization index is ≥2, respectively. Instead of the 1mg DST test, we performed the 24-hour urine cortisol levels dosage to exclude patients with cortisol co-secretion from undergoing AVS. Therefore, we did not measure other metabolites such as metanephrines for index correction beyond cortisol. APAs were verified via histopathology and immunohistochemical analysis (CYP11B2 staining positive). The adrenal tissues were screened for somatic mutations in the hot spot regions of KCNJ5, ATPase, CTNNB1, and CACNA1D. Clinical and biochemical success after adrenalectomy for unilateral PA was assessed using the Primary Aldosteronism Surgical Outcome (PASO) criteria (21). Complete clinical success was defined as achieving normalized BP (<140/90 mmHg) without use of anti-hypertensive medication after 6 months of follow-up. Normalized ARR and absence of hypokalemia were classified as complete biochemical success. All patients provided written informed consent, and the procedure received approval from the local ethics committee.

### Patients follow up

We followed up the patients 1 month, 1 year, and 5 years after surgery to assess outcomes, including antihypertensive requirement, BP, eGFR, and levels of potassium, aldosterone, and renin. We calculated eGFR using the CKD-Epidemiology Collaboration equation (22). Renal function deterioration was defined as a decrease in eGFR at 1 month postoperatively ≥30% of the preoperative eGFR. CKD was defined as an eGFR of <60 mL/min/1.73 m^2^.

### Genotyping

Genomic DNA was extracted from APAs according to the standard methods. Sequences of KCNJ5, ATP1A1, ATP2B3, CTNNB1, and CACNA1D were amplified using primers described previously (17, 23–25). Sanger sequencing of the purified polymerase chain reaction products was analyzed using an Applied Biosystems 3730xl DNA Analyzer.

### Statistical analyses

Continuous variables are expressed as mean ± standard deviations or medians with interquartile ranges, as appropriate. Categorical variables are presented as counts and percentages (*n* [%]). To compare continuous parameters, we used Student’s t-test or one-way analysis of variance, depending on the data distribution. For categorical variables, comparisons were made using the χ^2^ or Fisher exact test, as appropriate. Pearson’s correlation analysis was performed to examine the relationship between changes in BP and eGFR. Univariate and multivariate analyses were conducted to identify the risk factors for postoperative renal function deterioration. Statistically significant was set a *P*-value of <0.05. We used SPSS Statistics 26.0 and GraphPad Prism 9.0 for all statistical analyses.

## Results

### Baseline characteristics of patients

Of 218 patients with PA, *KCNJ5* somatic mutations were observed in 164 patients. The *ATP1A1* (L104R) somatic mutation was detected in four APAs, and the *ATP2B3* (p. 422-426del) somatic mutation was detected in two APAs. Additionally, the *CACNA1D* (G403R) somatic mutation was identified in one APA, and 46 cases presented with the WT. We analyzed the clinical features of 164 patients with *KCNJ5* mutation and 46 patients with *KCNJ5*-WT mutation. The patients were divided into three groups according to their age at PA diagnosis. The baseline clinical features of study patients are presented in **Table 1**. In total, 74 patients were <40 years old (35.2%), 102 (48.6%) were 40–60 years old, and 34 (16.2%) were ≥60 years old. There were significant differences in hypertension duration, diastolic blood pressure (DBP), percentage of *KCNJ5* mutation, and eGFR among the three groups. Patients aged <40 years had a shorter duration of hypertension, higher DBP, and higher eGFR than patients in the other age groups. In our study, the prevalence of *KCNJ5* mutation was 75.2%. It was highest in patients aged <40 years (83.8%) and lowest in patients aged ≥ 60 years (58.8%). We observed no significant differences in the percentage of sex, body mass index (BMI), systolic blood pressure (SBP), plasma aldosterone concentration (PAC), urinary aldosterone level, plasma renin activity, serum sodium and serum potassium levels, or prevalence of hypokalemia among the three groups. Although not statistically significant, SBP was relatively higher in patients aged ≥60 years than in patients in the other age groups.

**Table 1:** Baseline clinical characteristics of patients.

	<40 (n=74)	40–60 (n=102)	≥60 (n=34)	*P*
Age (years)	34 (31–37)^#,****^	51 (47–55)^$,****^	64 (61–67)^&,****^	<0.0001
Female (n, %)	38 (51.4)	45 (44.1)	12 (35.3)	0.2829
HT duration (years)	1.5 (0.5–4)^#,****^	8 (4–13)^$,****^	15 (10–20)^&,****^	<0.0001
BMI (kg/m^2^)	24.5 ± 4.1	24.0 ± 2.8	23.7 ± 2.7	0.4451
SBP (mmHg)	146.5 (137–156)	141.5 (133–157)	154 (140–160)	0.0583
DBP (mmHg)	90 (83–97)^#,*^	85 (80–94)	84 (79–90)^&,***^	0.0025
PAC (pg/mL)	314.6 (219.8–416.5)	296.9 (218.8–446.6)	345.8 (235.8–489.7)	0.6119
Urinary aldosterone (μg/24 h)	23 (17.3–72.5)	21.7 (16–67.6)	19.3 (13.6–30.6)	0.2835
PRA (ng/mL/h)	0.28 (0.14–0.58)	0.29 (0.15–0.67)	0.28 (0.15–0.66)	0.9436
Serum Na^+^ (mmol/L)	141 (134.8–143)	141 (140–143)	142.5 (141–144)	0.0547
Serum K^+^ (mmol/L)	3.16 ± 0.4	3.17 ± 0.4	3.2 ± 0.6	0.9445
Hypokalemia (n, %)	63 (85.1)	78 (76.5)	30 (88.2)	0.1853
KCNJ5 mutation n (%)	62 (83.8)	82 (74.5)	20 (58.8)^&,**^	0.0198
eGFR (mL/min/1.73 m^2^)	115.8 (107.6–120.2)^#,****^	101 (86.8–107.7)^#,****^	86.8 (70.5–97.8)^&,****^	<0.0001

Values are expressed as means ± standard deviations, medians (interquartile ranges), or numbers (%). HT, hypertension; BMI, body mass index; SBP, systolic blood pressure; DBP, diastolic blood pressure; PAC, plasma aldosterone concentration; PRA, plasma renin activity; eGFR, estimated glomerular filtration rate. ^#^<40 vs. 40–60, ^$^40–60 vs. ≥60, ^&^<40 vs. ≥60, ^****^ *P*<0.0001, ^***^ *P*<0.001, ^**^ *P*<0.01, ^*^ *P*<0.05.

### Outcomes after adrenalectomy

According to the PASO criteria, 43 (58.1%) patients aged <40 years, 24 (23.5%) patients aged 40–60 years, and 1 (2.9%) patient aged ≥60 years achieved complete clinical success (**Supplementary Table 1**). The analysis revealed significant differences in complete clinical success among the three groups (*P*<0.0001), with the <40 years old group having the highest success rate. Conversely, no significant differences in complete biochemical success were found among the groups (*P*=0.9169). All three groups exhibited a high prevalence of complete biochemical success. After adrenalectomy, none of the patients in the <40 years old group developed hyperkalemia. However, five of 102 (4.9%) patients aged 40–60 years and five of 34 (14.7%) patients aged ≥60 years presented with hyperkalemia (**Supplementary Table 1**).

### Longitudinal changes in renal function and BP after adrenalectomy

**Figure 1:**
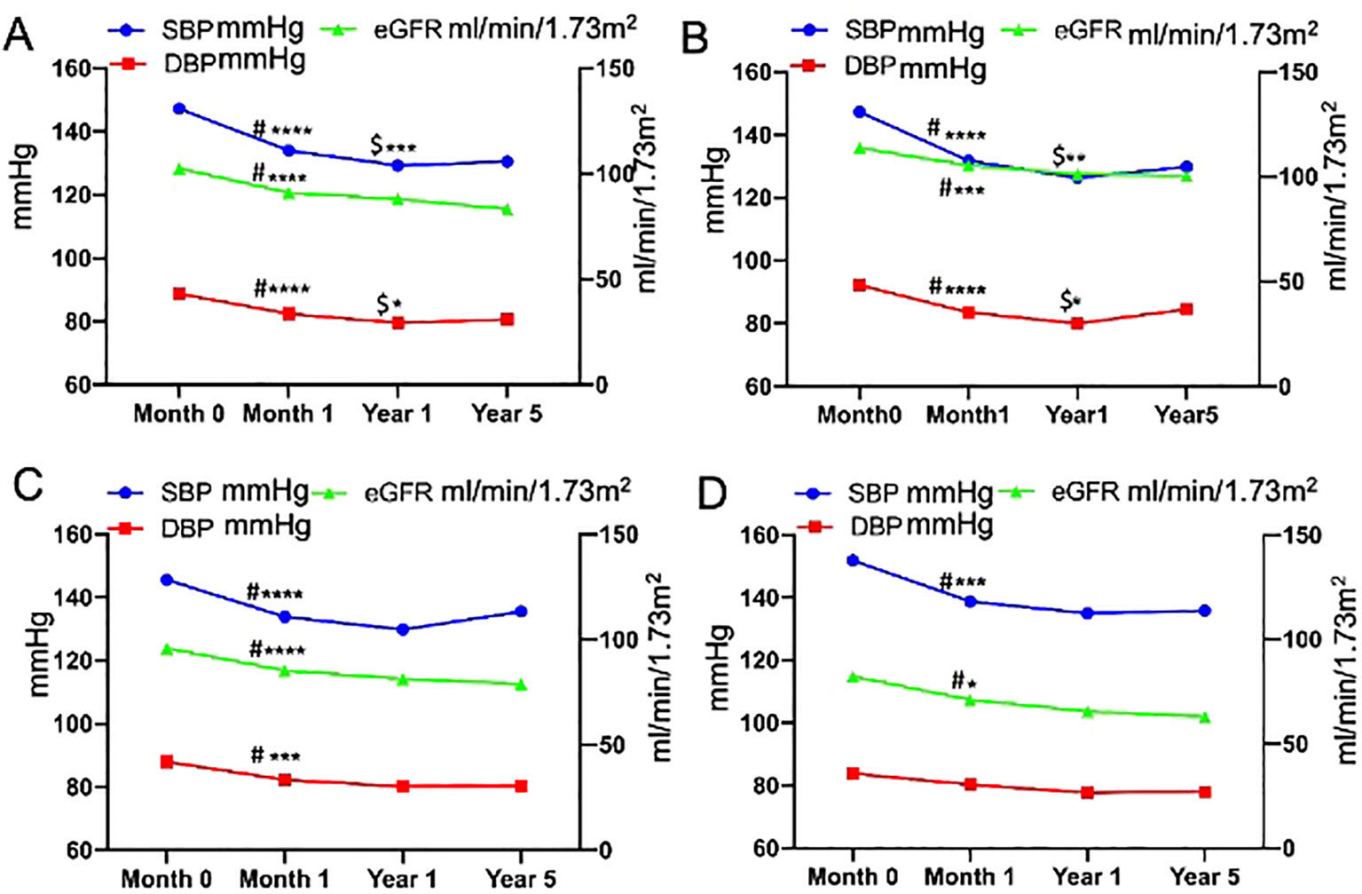
Longitudinal changes in blood pressure and estimated glomerular filtration rate before and after adrenalectomy [**(A)**, all patients; **(B)**, patients aged <40 years; **(C)**, patients aged 40–60 years; and **(D)**, patients aged ≥60 years]. ^#^Month 0 vs. Month 1, ^$^Year 1 vs. Month 1, ^****^P<0.0001, ^***^P<0.001, ^**^P<0.01, ^*^P<0.05.

Changes in BP and eGFR before and after adrenalectomy are shown in **Figure 1**. The SBP, DBP, and eGFR of all patients decreased 1 month after surgery (*P*<0.0001, respectively). SBP and DBP were further reduced at 1-year post-surgery compared to that at 1 month after adrenalectomy, whereas no significant change was found in eGFR at 1 year versus at 1 month. SBP and DBP slightly increased, and eGFR continued to decline at 5 years postoperatively, whereas there were no significant differences compared with those at 1 year postoperatively.

**Figure 2:**
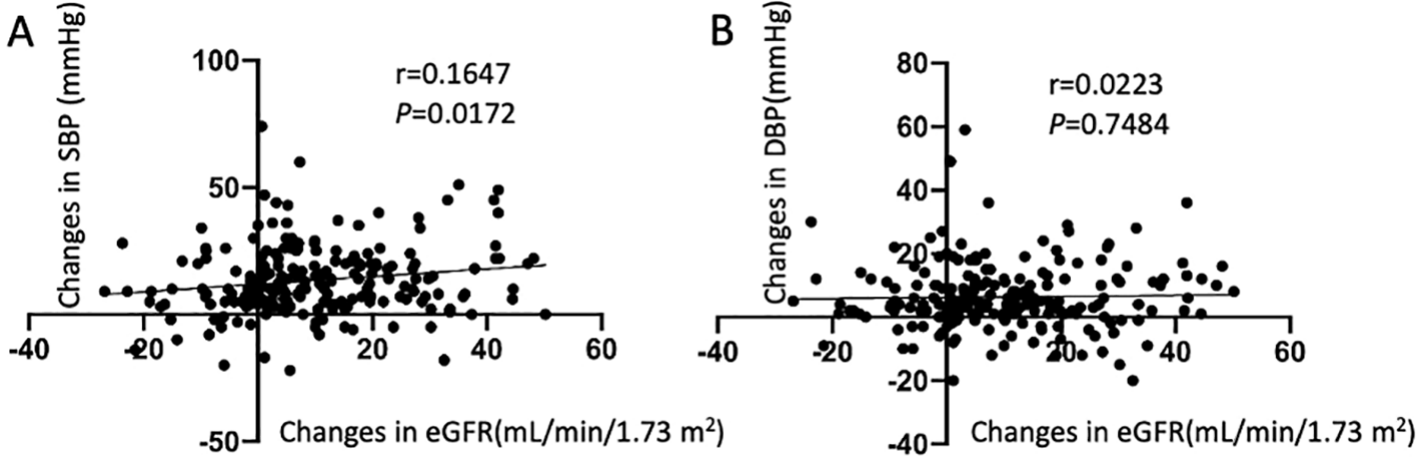
Correlation between the changes in estimated glomerular filtration rate (eGFR) and the changes in blood pressure. **(A)** Relationship between changes in systolic blood pressure and eGFR; **(B)** Relationship between changes in diastolic blood pressure and eGFR.

In all patients, the change in SBP (preoperative SBP– 1-month postoperative SBP) was positively correlated with the change in eGFR (preoperative eGFR– 1-month postoperative eGFR) (**Figure 2**), and the eGFR declined along with the decrease in BP. There were no significant differences in the changes in BP and eGFR among the three age groups, either in the short- or long-term after adrenalectomy (**Table 2**). Only the change in SBP in patients aged <40 years showed a significantly positive correlation with the change in eGFR (**Supplementary Figure 1**).

**Table 2:** Short- and long-term decrease in blood pressure and eGFR in different age groups.

	<40	40–60	≥60	*P*
SBP (mmHg)				
Short-term Long-term	3.8 ± 14.1 20.5 ± 22.3	3.1 ± 17.5 14.2 ± 19.7	0.9 ± 17.8 13 ± 18.2	0.8315 0.2906
DBP (mmHg)				
Short-term Long-term	0.7 ± 12.7 11.3 ± 17	2.8 ± 12.1 7.5 ± 12.6	2.4 ± 9.8 6.7 ± 7.3	0.9048 0.3567
eGFR (mL/min/1.73 m^2^)				
Short-term Long-term	9.4 ± 15.7 14.8 ± 14.8	9.9 ± 16.5 15 ± 15.4	11.1 ± 15.9 17.6 ± 15.9	0.9207 0.7682

Short-term: pre-operation–1-month post-operation; long-term: pre-operation–5-years post-operation. eGFR, estimated glomerular filtration rate; SBP, systolic blood pressure; DBP, diastolic blood pressure.

Univariate logistic regression analysis demonstrated that *KCNJ5* mutation status was significantly negatively correlated with the occurrence of CKD (odds ratio [OR], 2.825; 95% confidence interval, 1.123–7.110; *P*=0.027) (**Table 3**). However, it did not correlate with the changes in SBP, DBP, and eGFR, respectively. No significant differences were observed in CKD occurrence among the three age groups.

**Table 3:** Relationship between the KCNJ5 mutation status and changes in blood pressure and renal function.

Variable	Univariate model	
	OR (95% CI)	*P* value
△ SBP (mmHg)	0.999 (0.976–1.023)	0.939
△ DBP (mmHg)	1.016 (0.983–1.050)	0.349
△ eGFR (mL/min/1.73 m^2^)	1.000 (0.979–1.022)	0.992
CKD (eGFR<60 mL/min/1.73 m^2^)	2.825 (1.123–7.110)	0.027

△ SBP, changes in systolic blood pressure between pre-op and 1-month post-op; △ DBP, changes in diastolic blood pressure between pre-op and 1-month post-op; △ eGFR, changes in diastolic blood pressure between pre-op and 1-month post-op; OR, odds ratio; CI, confidence interval; eGFR, estimated glomerular filtration rate; SBP, systolic blood pressure; DBP, diastolic blood pressure; CKD, chronic kidney disease.

After adrenalectomy, the proportion of patients aged ≥60 years who experienced a drop in eGFR of >30% was higher than that in the younger groups, although this difference was not statistically significant (**Supplementary Figure 2**). The proportion of eGFR decreased by >30% or eGFR<60 mL/min/1.73 m^2^ after adrenalectomy in patients with *KCNJ5*-WT was higher than that in patients with *KCNJ5* mutation. Additionally, the percentage of patients with postoperative hyperkalemia was also higher in patients with *KCNJ5*-WT than in patients with *KCNJ5* mutation (**Table 4**).

**Table 4:** Changes in renal function in patients with KCNJ5-WT and KCNJ5 mutations.

Characteristic	KCNJ5 wild type (n=46)	KCNJ5 mutant (n=164)	*P*
eGFR decreased >30%, n (%)	11 (23.9)	19 (11.6)	0.035
eGFR<60 mL/min/1.73 m^2^(%)	9 (19.6)	13 (7.9)	0.027
Hyperkalemia, n (%)	5 (10.8)	5 (3.0)	0.028

eGFR, estimated glomerular filtration rate.

### Risk factors for postoperative renal function deterioration

Characteristics that were significant in the univariate analysis (*P*<0.1) were included in the multivariate analysis. Univariate logistic regression analysis showed that male sex, age, HT duration, preoperative SBP, PAC, and *KCNJ5*-WT were significantly associated with renal function deterioration (a decrease in eGFR at 1 month postoperatively ≥30% of the preoperative eGFR). Multivariate analysis revealed that HT duration, preoperative SBP, and PAC were predictors of renal function deterioration after adrenalectomy (**Table 5**).

**Table 5:** Risk factors for postoperative renal function decline 1 month after adrenalectomy.

Variable	Univariate model		Multivariate model	
	OR (95% CI)	*P* value	OR (95% CI)	*P* value
Male sex	2.135 (0.927–4.915)	0.075	1.611 (0.615–4.225)	0.332
Age	1.035 (1.001–1.070)	0.043	0.996 (0.947–1.049)	0.891
HT duration	1.081 (1.034–1.131)	0.001	1.085 (1.014–1.160)	0.018
BMI	1.111 (0.992–1.244)	0.07	1.158 (1.000–1.341)	0.050
SBP	1.033 (1.008–1.057)	0.008	1.037 (1.009–1.066)	0.009
DBP	1.016 (0.984–1.049)	0.339		
Serum K^+^	0.775 (0.335–1.792)	0.551		
PAC	1.001 (1.000–1.003)	0.045	1.002 (1.000–1.003)	0.031
PRA	1.159 (0.732–1.824)	0.524		
eGFR	0.994 (0.974–1.013)	0.513		
*KCNJ5*	0.432 (0.189–0.988)	0.047	0.740 (0.277–1.973)	0.547

HT, hypertension; BMI, body mass index; SBP, systolic blood pressure; DBP, diastolic blood pressure; PAC, plasma aldosterone concentration; PRA, plasma renin activity; eGFR, estimated glomerular filtration rate; OR, odds ratio; CI, confidence interval.

## Discussion

In this study, we presented the longitudinal changes in eGFR before and after adrenalectomy in patients with PA, focusing on different age groups. We also identified the risk factors for postoperative eGFR decline in these patients. Furthermore, our findings suggest that somatic *KCNJ5* mutation may confer a protective effect against renal dysfunction after adrenalectomy.

Previous studies have reported that adrenalectomy reduced long-term mortality, major cardiovascular events, and incidence of congestive heart failure (26). Additionally, it also lowers incident atrial fibrillation in PA patients at long term (27) and improves metabolic outcomes (28). But studies on long-term postsurgical renal outcomes are scarce. Patients with PA have a higher risk of renal impairment and a greater prevalence of proteinuria than do patients with essential hypertension (EH) (29, 30). Sechi et al. (31) reported a decrease in eGFR and albuminuria during the initial 6-month treatment (surgical or medical treatment) in both the PA and EH patients, with a significantly greater change observed in patients with PA than in patients with EH. The subsequent rate of decrease in glomerular filtration was comparable between the two groups, whereas albuminuria did not progress further during the remainder of the follow-up period, indicating that renal dysfunction may be partially reversible with PA treatment. These findings underscore the importance of early PA identification for the effective prevention of long-term renal complications. Our findings indicate that eGFR decreases significantly during the initial 1-month period after adrenalectomy compared with that before surgery in all patients. In patients with PA, long-term aldosteronism leads to high blood volume, which increases BP and glomerular filtration. Excess aldosterone helps maintain eGFR and masks underlying structural renal damage. Accordingly, renal impairment can be revealed once aldosterone levels dramatically decrease after adrenalectomy (13, 32). We further found that a decrease in postoperative BP positively correlated with a decrease in eGFR, particularly in younger patients. However, no further significant reduction was noted in the long-term follow-up (1 and 5 years) after adrenalectomy across all age groups. We conclude that the underlying mechanisms may be associated with rapid aldosterone reduction, postoperative hypovolemia, an abrupt drop in BP, and renal hypoperfusion after adrenalectomy. Over time, all aforementioned factors achieve a new equilibrium. Considering these observations, monitoring postoperative BP changes is crucial. Considerations include discontinuing or reducing antihypertensive drugs 1–2 days pre-surgery, increasing postoperative fluid intake, and adopting a sodium-rich diet to prevent excessive BP drops, which may mitigate renal function decline.

Our study also revealed that the postoperative complete biochemical success rates were comparable among all three groups. In contrast, complete clinical success rates are lower among older patients than among younger and middle-aged patients, which is largely attributed to prolonged hypertension duration and existing vascular lesions in older patients. However, eGFR levels in older patients did not decline sharply after surgery compared with those in younger cohorts, suggesting the potential benefits of surgery in this demographic.

Additionally, we observed postoperative hyperkalemia in middle-aged (5/102) and older (5/34) patients, but not in younger patients. Although the incidence of postoperative hyperkalemia is relatively low in middle-aged and older patients with PA, it remains a concern. Close monitoring of renal function and serum potassium levels immediately after surgery is essential. We also discontinue all antihypertensive agents, including spironolactone and potassium-sparing antihypertensive agents after surgery and add calcium-channel blocker if needed, besides, we encourage patients to follow a low potassium diet for short-term. Long-term changes in BP may be influenced by age, lifestyle, and medication.

Recently, several studies have investigated the risk factors for postoperative renal dysfunction in patients undergoing adrenalectomy for PA, including preoperative eGFR, urinary albumin excretion, ARR, age, serum potassium level, BMI, and hypertension duration (14, 33–35). Kim et al. regarded a high preoperative eGFR is identified as a risk factor for postoperative renal dysfunction, whereas a low preoperative eGFR is associated with a likelihood of developing into CKD postoperatively (34, 36). Yoshioka et al. (37) reported that age was a critical predictor of kidney dysfunction. They found that adrenalectomy might result in a risk of renal impairment in patients with PA aged ≥50 years. However, our findings indicate that preoperative eGFR and age are not primary determinants of postoperative renal function decline. Haze et al. (38) reported that higher SBP 6 months after PA treatment was associated with a higher risk of renal impairment over time, independent of the BP levels before treatment. Contrary to these findings, our data revealed that higher preoperative SBP was associated with lower postoperative eGFR. Our findings are partly consistent with those of previous studies demonstrating that a longer duration of hypertension, higher preoperative SBP, and elevated PAC levels are significant risk factors for kidney function deterioration after surgery. In addition, in our study, the prevalence of *KCNJ5* mutation was 75.2%, which is higher than that reported in Western countries. We also observed that the presence of somatic *KCNJ5* mutation was inversely correlated to the postoperative occurrence of CKD, suggesting a protective effect of *KCNJ5* mutation against CKD development after unilateral adrenalectomy. Yoshioka et al. (37) also reported that patients with APAs aged ≥50 years who progressed to CKD showed a higher incidence of *KCNJ5* mutation rates (75%). However, in that study, the deterioration of CKD was defined as progression to the CKD category, and the cutoff age was 50 years, which is different from the age classification and definition of postoperative renal function decrease used in our study. In addition, the sample size of their study was relatively small. KCNJ5 mutations are thought to be associated with florid PA phenotype. After unilateral adrenalectomy, the effects of KCNJ5 mutations are abolished, leading to the normalization of potassium levels and dramatically decreasing blood volume. These changes subsequently reduce renal workload and mitigates tubular damage, thereby protecting renal function in the postoperative period. In addition, patients with KCNJ5 mutations are younger, who have a shorter hypertension duration and less severe vascular remodeling. In contrast, KCNJ5-WT patients exhibit the opposite characteristics. Moreover, older patients with PA usually have concomitant EH, which negatively impacts renal function postoperatively. These may be the reasons why KCNJ5 mutations have a protective effect on postoperative kidney function.

Middle-aged and older patients can benefit from unilateral adrenalectomy, despite exhibiting slightly lower pre- and postoperative eGFR levels than younger patients. Therefore, surgical treatment remains advisable for older patients with PA if unilateral PA is confirmed using AVS. Considering the higher incidence of postoperative hyperkalemia in middle-aged and older patients, immediate postoperative follow-up is recommended.

Our study has some limitations. First, this was a long-term retrospective study in which other factors, such as lifestyle, the occurrence of diabetes, or other types of kidney disease, could potentially influence BP and eGFR during follow-up. Second, we assessed changes in renal function solely using eGFR and did not monitor albuminuria. Future studies should include monitoring the urinary albumin/creatinine ratio and 24-h urinary protein quantity during postoperative follow-up. Third, in our study, the follow-up time points were set at 1 month, 1 year, and 5 years after surgery, which may have caused us to miss the rebound in eGFR that occurs in the 1-3 months post-surgery.

In conclusion, our study provides evidence that unilateral adrenalectomy is beneficial for patients with PA, showing favorable outcomes in terms of both biochemical and clinical success, irrespective of age. Preoperative SBP, PAC, and hypertension duration were significant predictors of postoperative renal function impairment. Additionally, *KCNJ5* mutation appears to offer protection against CKD in patients with PA after unilateral adrenalectomy. Attention should be paid to the occurrence of postoperative renal function impairment and hyperkalemia, especially in older patients. These insights are expected to provide valuable guidance for the management of patients with PA.

## Data Availability

The original contributions presented in the study are included in the article/**Supplementary Material**. Further inquiries can be directed to the corresponding author.
