# Supplementary material for: Chronological outcomes of renal function after adrenalectomy in patients with primary aldosteronism across age groups
Source: Front Endocrinol (Lausanne). 2024 Nov 7;15:1467742. doi: 10.3389/fendo.2024.1467742 (PMC11578701; doi:10.3389/fendo.2024.1467742)
Supplement: Supplementary file 1 [file DataSheet1.docx]

Supplemental Table 1. Outcomes after adrenalectomy

|  | <40 (n=74) | 40–60 (n=102) | ≥60 (n=34) |
| --- | --- | --- | --- |
| Complete clinical success (%) | 58.1 | 23.5 | 2.9 |
| Complete biochemical success (%) | 93.2 | 92.1 | 94.1 |
| Hyperkalemia (%) | 0 | 4.9 | 14.7^**^ |

^**^*P*<0.01 <40 vs. >60


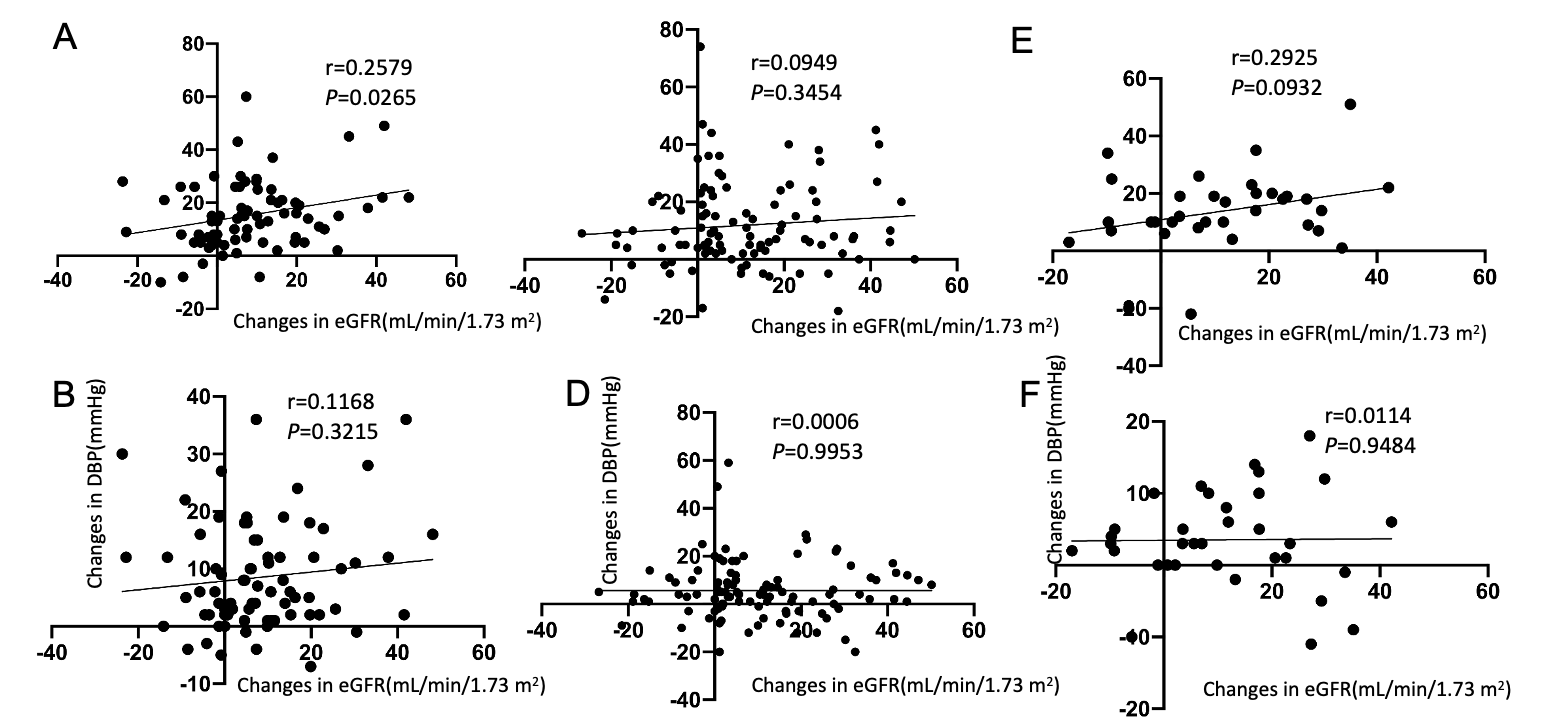


Supplemental Figure 1. Relationship between changes in estimated glomerular filtration rate (eGFR) and blood pressure at different ages. Relationships between the changes in eGFR and changes in systolic blood pressure or diastolic blood pressure, (A–B) patients aged <40 years, (C–D) patients aged 40–60 years, (E–F) patients aged ≥60 years.


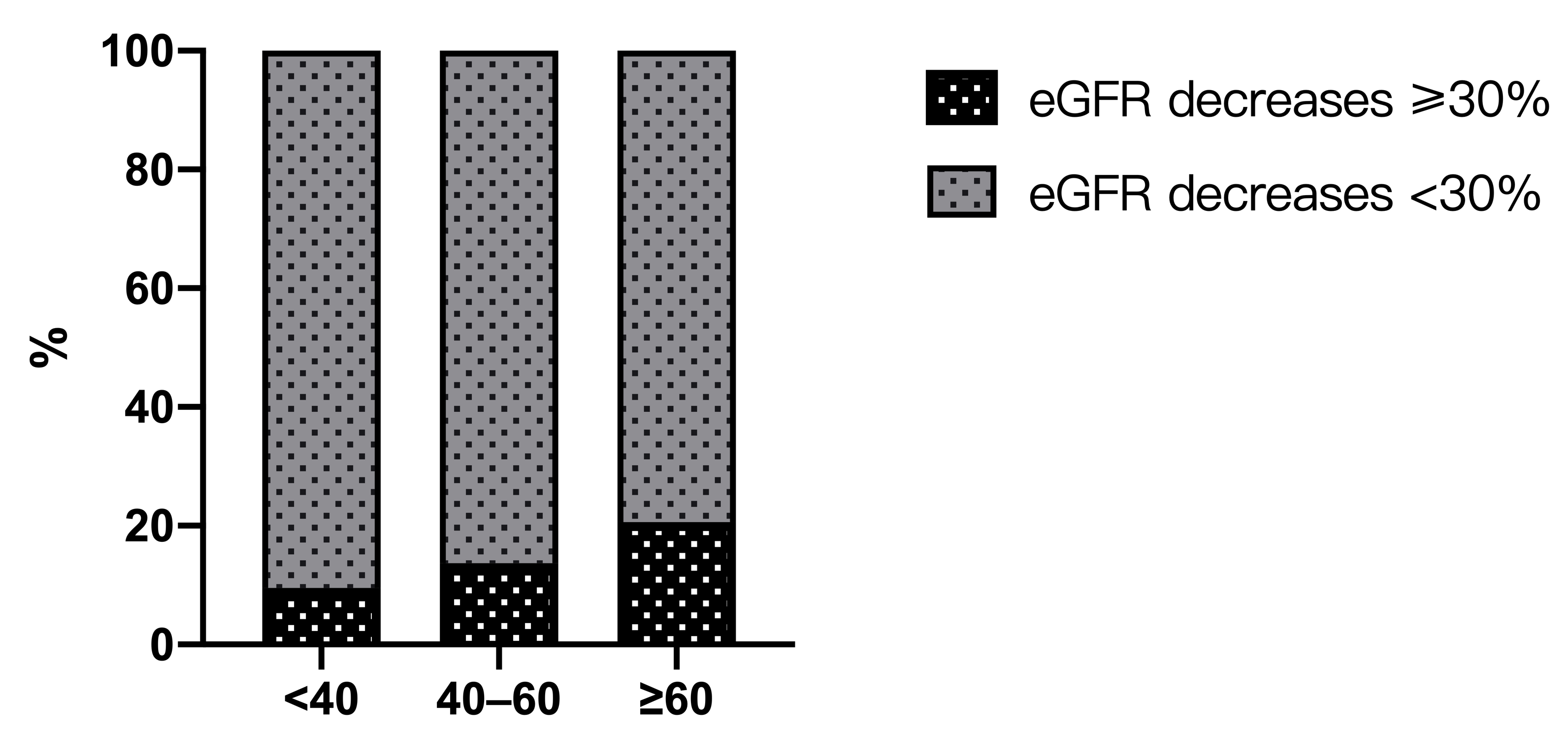


Supplemental Figure 2. Prevalence of estimated glomerular filtration rate (eGFR) decreased by >30% after adrenalectomy in all subgroups.

Prevalence of eGFR decrease by >30% after adrenalectomy was higher in patients aged ≥60 years than in the other age groups.
